# Diabetic neuropathy and the sensory apparatus “meissner corpuscle and merkel cells”

**DOI:** 10.3389/fnana.2014.00079

**Published:** 2014-08-14

**Authors:** Salma Alsunousi, Husnia I. Marrif

**Affiliations:** ^1^Department of Histology, Faculty of Medicine, Benghazi UniversityBenghazi, Libya; ^2^Department of Pharmaceutical Sciences, Princess Noura UniversityRiyadh, Saudi Arabia

**Keywords:** diabetic neuropathies, xons, meissner corpuscle, polyneuropathy, polyneuropathies

## Introduction

As diabetes progresses, a myriad of symptoms and signs appear in patients. Of these, distal symmetric peripheral neuropathy is one of the most devastating and common complications (Vinik et al., 2013). Etiology of diabetic neuropathy is complicated, and not fully understood. Yet strong evidence points at hyperglycemia and glycemic control as the starting point of the diabetic complications. For reference and details please refer to Farmer et al. (2012).

The complexity of neuropathology can be clearly seen in the range of patient complains related to change in motor, autonomic and sensory functions. A key investigation of the underlying neuropathology associated with sensory polyneuropathy is the study by Shun et al. (2004). In our opinion, it demonstrates a superb visualization of clinically case of neuropathology. In this opinion article, we dissect Shun's article and scrutinize the findings.

## Classification of diabetic neuropathology

**Figure 1:**
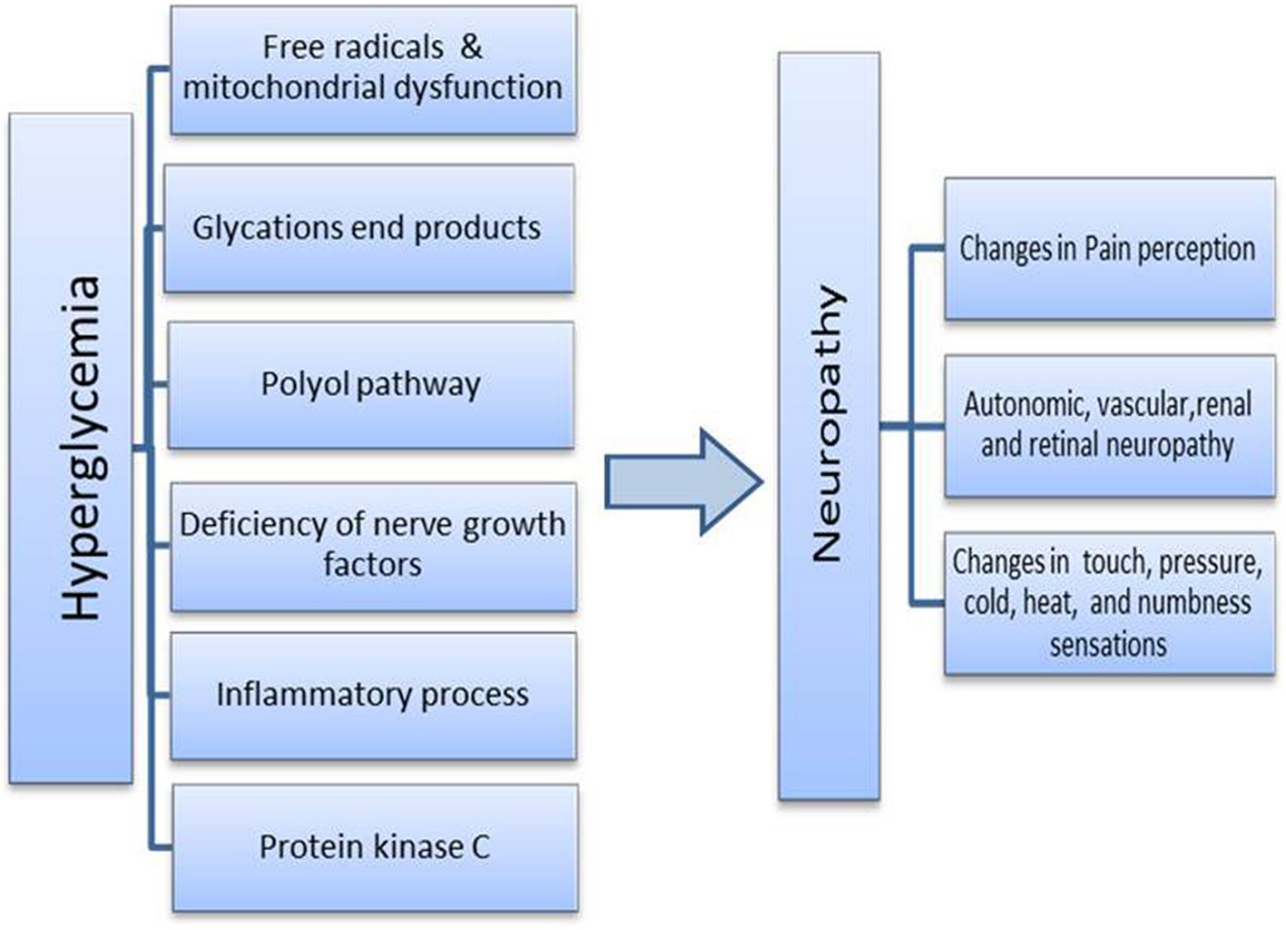
**Possible trigger and mechanism involve in development of diabetic neuropathy**.

Neuropathology associated with diabetes is unequivocally an axonal issue. It can affect autonomic, myelinated motor, myelinated fine, or unmyelinated somatic sensory axons (Said, 2007). In brief, diabetes neuropathy can be observed as symmetric polyneuropathy of axons or focal asymmetric neuropathy associated with lesion and inflammation (Farmer et al., 2012). Different pathways are implicated in the development and progress of neuropathy (Figure 1). However, it is pivotal to bear in mind that the real culprit behind all the neuropathology is the state of hyperglycemia (Du et al., 2000).

Categorically, the proposed mechanistic processes involved in the etiology are: Polyol pathway, glycations end products, protein kinase C, oxidative/ free radicals process and mitochondrial dysfunction, inflammatory process and deficiency of nerve growth factors. It is not a single cause, rather a mayhem of metabolic and deleterious cellular processes. The overall pathology could also be extended to tangible changes in vascular structure. For a review, please refer to Farmer et al. (2012). Clinically, the most common form of diabetic polyneuropathy is usually observed in the lower limbs, in the extended long axons. As the degenerative process reaches the upper body short axons, symptoms appear in hands and finger tips (Said and Krarup, 2013).

The symptoms can include: paresthesia (numbness), allodynia, hyperalgesia (lower pain threshold), and dysesthesia (loss of pain sensations). Symptoms usually start in the long axons of the lower limbs and progress upward to the short axons of the hands and fingers. It is in fact also named “fiber length dependent pattern” as it is associated with the length of axons (Said, 2007). The progress of neuropathy seems to have different path and mechanism in insulin dependent and type two diabetes, there are some evidence in literature which suggest that in prediabtic type two patients up normal unmyelinated c fiber changes preceding large myelinated fiber changes (Myers and Peltier, 2013). The presence of autonomic and vascular neuropathy, ischemia, inflammation, and infection can lead to the grim point of loss of the patient's limb.

## Mechanisms of nerve injury

The two most prominent complaints in diabetic neuropathy are peripheral pain (nociception) and change in touch which includes; numbness, cold or heat sensing (Bierhaus et al., 2004). Two types of sensory axons which carry these kinds of signals are the myelinated subtle fibers or axons of A delta sensory type and unmyelinated fine C fibers (Christianson et al., 2007). In a plethora of publications, one study in fact opens the argument of how diabetes manipulates heat, pressure, and pain sensation and their thresholds. A study from the laboratory of Hsieh in 2004 elegantly and visually describes the possible changes in neuronal sensory structure of skin layers in diabetic neuropathy. For details, please refer to Shun et al. (2004).

## The visual art

The study by Hsieh's laboratory in 2004 included diabetic patients with sensory symptoms including foot with graded stocking pattern. The researchers quantified sensory response to hot, cold, vibration, and kinaesthetic stimuli. They used a battery of tests including skin biopsy, immunohistochemistry, thermal sensory analysis, and nerve conduction studies. For details, please refer to Shun et al. (2004).

The study reports that in comparison to normal subjects, diabetic patients had abnormal thresholds to warm and cold stimuli; 81.6% of diabetic patients had elevated warm threshold and a change of 57.9% in cold threshold. The study also shows a higher vibration threshold of about 63.2% and a significant reduction in nerve conductance speed in diabetic patients as compared to normal subjects.

In our opinion, the striking result in this study was the skin biopsy and histology work. It revealed a complete change in skin layer topography. The integumentary system contains sensory axons which conduct different sensory stimuli to the spinal cord. The array of conductance includes touch, pressure, cold and heat, and pain, and all are mediated by small c fibers and or fine myelinated axons (Bril and Buchanan, 2006; Obrosova et al., 2010).

In the study performed by Shun, two key observations are reported. First, in the epidermis layer of diabetic patients, a marked reduction of nerve density and complete denervation in some biopsies were found. In the dermis layer, they reported that in the control subjects, nerves were bundled up in a network surrounding the layer while in the diabetic patients, the nerves became fragmented. Please refer to Figure 2.

First, considering conflicting animal model studies, this is a superb observation in human diabetic tissue. Second, it provides a window to the end point of neuropathy regardless of the mechanism of destruction during diabetes neuropathy. Third, the study opened the question about the fate of mechanoreceptors and their role in degenerative diabetes, this article will focus on specific structure but overall mediators of mechanoreceptors referee to Table 1.

## Sensory machineries

Morphological studies of diabetic neuropathy have provided some evidence that certain machinery used by the nervous system in sensing are damaged and their function is altered by diabetes. Mechanoreceptors machineries found in skin include Meissners's corpuscles, Pacinian, Lanceolate ending and Merkel cells and others. As most of the complications of diabetes occurs in distal axons enervated glabrous skin, we will only focus on Meissners's corpuscles and Merkel cells as the others are mainly associated with haired skin. For an overall view of mechanoreceptor structures found in human refer to Table 1.

## Meissners's corpuscles

These are tactile afferent mediating structures found within dermal papillae. They are conical shaped protrusions of the dermis into the epidermis layer, with the main structure reaching the surface of the skin (Fleming and Luo, 2013). They have an intriguing structure which encompasses all the clues to construe the etiology of neurodegenerative process. The corpuscles composed at the center of stacks of lamella are derived from Schwan cells surrounded by thin fibroblast capsules. The structure is innervated by myelinated AB sensory fiber which loses its myelination after traveling through the bottom third of the corpuscle, and by unmyelinated varicose afferent and non-varicose C fibers (Paré et al., 2001). The AB fibers are low threshold sensing fibers which project to the dorsal horn of the spinal cord.

As summarized by Fleming and Luo (2013), those oval machinery shaped structures are described as having very low threshold for sensory stimuli. They respond to low stimulation as slow as 5 Hz (Palmer and Gardner, 1990) and they respond to an indentation of skin of less than 10 um (Iggo and Ogawa, 1977). For details, please refer to the review by Fleming and Luo (2013).

Shun's study of diabetic skin biopsy in 2004 reported two key observations. In some samples, a complete epidermis denervation was observed, and in deep dermis, researchers reported a change in neuronal structure from the strong bundled cables of network to a scattered pattern of disease state. As Meissners's corpuscles are a part of sensory apparatus which send signals through the nerve plexus and cables to the spinal cord, would we still expect to have normal sensory processes if we cut or reroute cables?

## The merkel cells

Another intriguing structure in the epidermis layer of the skin are the Merkel cells. They are part of the mechanoreceptors' neuronal cells. They respond to touch stimuli and are found in the epidermal layer of fingers and feet tips. For more details, please refer to the review article (Fleming and Luo, 2013). Merkel cells are innervated by long axons and they form a network known as the Merkel cell neurotic complex. What has been reported in Shun's study is that neuronal axons in the dermis and epidermis were reduced. This observation alone suggests the loss of axons probably for both the Merkel cells as well as the Meissner's corpuscles.

Furthermore, the presence of a scattered neuronal bundle in the deep dermis was observed. These clues indicate the presence of an injury to axons, changes in neuronal guidance cues, or changes to growth cone and trophic factors. Growth cones are located at the tips of axons, which helps axons navigate their way to targets. Many molecules could be part of this picture. Netrins, Slits or others, for a review please refer to Nugent et al. (2012).

One of the hallmarks of diabetic peripheral neuropathy is loss or change of sensation to cold (threshold), heat, and touch. It's an organic damage of axons or structure connected to axons by the deleterious process of hyperglycemia. Many factors could be responsible for the insult and change in tissue topography, such as free radical damage, metabolic bi-products of cell glycolysis, edema, and inflammatory processes. We think Shun's paper has provided the first visual and pictorial evidence of the axonal degenerative process in human tissue by diabetes, a unique study which integrates the clinical agony with visual art.

### Recent views of diabetic neuropathy

An earlier European study in 16 European countries reported that almost 25% of type one diabetic patients developed painful diabetic neuropathy (Tesfaye et al., 1996). Current data provided a staggering numbers of diabetes and diabetic neuropathy in different countries. According to World Health Organization, global diabetes prevalence is approaching 5% worldwide (Zychowska et al., 2013). In the United States, the diabetic prevalence is estimated to be 23.1% in population of 60 years and older. The overall prevalence of painful diabetic neuropathy in the United Kingdom is about 21%, and the incidence is reported to be much lower in type one diabetic 13.4% in comparison to type two diabetic 21.5% (Abbott et al., 2011). In Korea, the prevalence was reported to vary from 13.1 to 61.8% (Won et al., 2014).

A very similar results were observed In developing countries, diabetic neuropathy estimate is about 24% (Katulanda et al., 2012). The above data, shows how significant is the prevalence of diabetes and its long term complications worldwide. Further to this point, it's also an indicator for how much is the burden of diabetic care on economy. Diagnosis and treatment of diabetic neuropathy is still a gray area. This filed lacks clear guidelines for diagnosis and treatment. Toward more accurate diagnosis, recently the European Federation of Neurological Studies recognized skin biopsy and nerve density procedure as sensitive measures of small fiber neuropathy (Lauria et al., 2010). Different techniques have been used to assess changes in neuropathy associated with diabetes and that includes: nerve conduction studies (for velocity and amplitude), monofilaments is used mostly for myelinated large fibers (assessment of pressure, vibration and touch perception), and skin biopsies. The latter followed by immunohistochemistry are used in assessment of unmyelinated fine C fibers. Other methods such as using neurological scoring systems are also valid tools in clinical research of diabetic neuropathy, for details please refer to Peltier et al., 2014.

It's worth mentioning that despite the significant information which could be obtained by skin biopsy, this rout is not a major rout used to confirm diagnosis of neuropathy (Younger, 2010). No cure is available for diabetic neuropathy and all that could be offered to a patient is different classes of drugs which are used for symptomatic management of diabetic pain or to slowdown the disease progress. As partial nerve injury might be at the core of the pathology, some very promising studies suggested the use of pluripotent stem cells to repair the damaged nerve (Mizukami and Yagihashi, 2014).

### Conflict of interest statement

The authors declare that the research was conducted in the absence of any commercial or financial relationships that could be construed as a potential conflict of interest.
