# Supplementary figures and images for: Diabetic neuropathy and the sensory apparatus “meissner corpuscle and merkel cells”
Source: Front Neuroanat. 2014 Aug 14;8:79. doi: 10.3389/fnana.2014.00079 (PMC4132297; doi:10.3389/fnana.2014.00079)

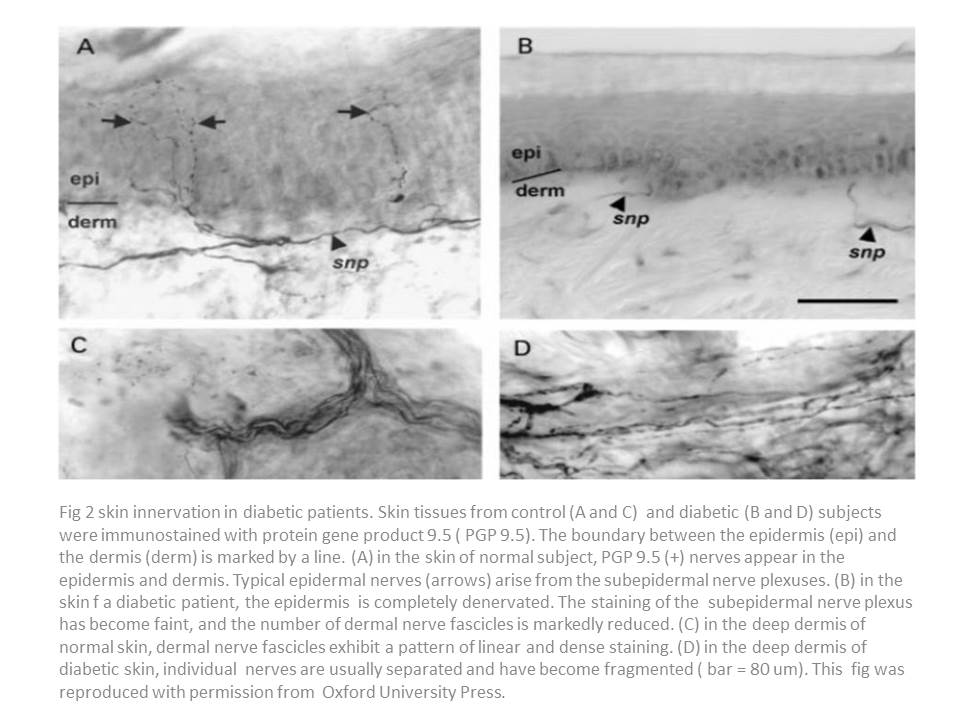

Supplement: Supplementary file 1 [file Presentation1.ZIP › Supp Figure.jpg]

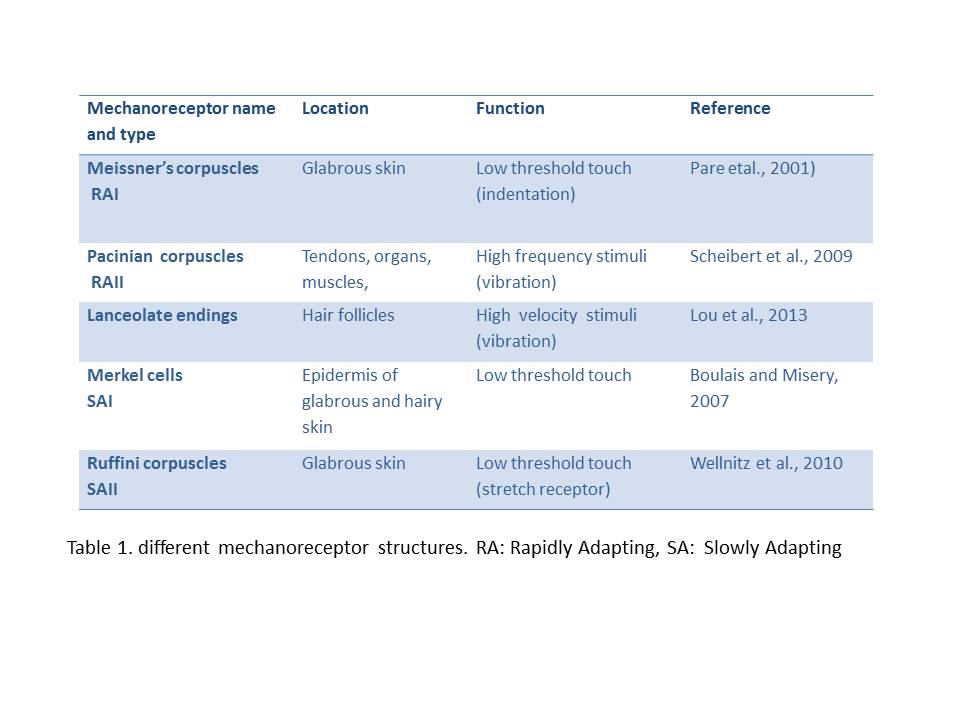

Supplement: Supplementary file 1 [file Presentation1.ZIP › Supp Table.jpg]
